# Photoinduced Charge Transport in a BHJ Solar Cell Controlled by an External Electric Field

**DOI:** 10.1038/srep13970

**Published:** 2015-09-10

**Authors:** Yongqing Li, Yanting Feng, Mengtao Sun

**Affiliations:** 1 Department of Physics, Liaoning University, Shenyang 110036, P. R. China; 2 Beijing National Laboratory for Condensed Matter Physics, Institute of Physics, Chinese Academy of Science, P. O. Box 603-146, Beijing, 100190, P. R. China; 3 State Key Lab of Molecular Reaction Dynamics, Dalian Institute of Chemical Physics, Chinese Academy of Sciences, Dalian 116023, China

## Abstract

This study investigated theoretical photoinduced charge transport in a bulk heterojunction (BHJ) solar cell controlled by an external electric field. Our method for visualizing charge difference density identified the excited state properties of photoinduced charge transfer, and the charge transfer excited states were distinguished from local excited states during electronic transitions. Furthermore, the calculated rates for the charge transfer revealed that the charge transfer was strongly influenced by the external electric field. The external electric field accelerated the rate of charge transfer by up to one order when charge recombination was significantly restrained. Our research demonstrated that photoinduced charge transport controlled by an external electric field in a BHJ solar cell is efficient, and ***the exciton dissociation is not the limiting factor in organic solar cells.***Our research should aid in the rational design of a novel conjugated system of organic solar cells.

Organic solar cells are a rapidly developing novel technology with environmental and economic^1^,^2^,^3^ advantages. Organic solar cells can be grouped into small organic molecule cells and polymer cells, the latter of which are a popular research area. A significant number of experimental and theoretical investigations have been performed to improve the photoelectric transformation efficiency of bulk heterojunction polymer solar cells. Donors and acceptors are important carriers in photoelectric transformation, and these carriers determine the photoelectric transformation efficiency^4^,^5^. Under light, electron-hole pairs can be created in donors. Under the influence of a built-in electric field, the electron-hole pairs separate and are transferred to the electrodes, where they are collected. In electric charge transfer, the electrons move to the cathode, while the holes move to the anode. Theoretical calculations play an important role in studying the relationship between the optical properties and the chemical structures of electronic donors and acceptors. Theoretical calculations are also critical for designing a novel and feasible donor-acceptor system. From the electronic structure perspective, donors and acceptors have a strong electronic coupling effect, which results in a low exciton binding energy. Quantum calculations can provide important information for the study of the photoinduced electric charge transfer mechanism in a donor-acceptor system. Recently, many organic solar cell materials with high photoelectric transformation efficiencies have been created, such as PCDPTBT/PC_61_BT^6^, PTB_7_/PC_71_BM^7^, PBDTT-DPP/PC_61_BM and PCPDT-BT/PC_71_BM^8^,^9^, for which the photoelectric transformation efficiencies are 6.7%, 7.4%, 8.6% and 10.6%, respectively.

Electric charge transfer theory has developed rapidly since the electric charge transfer phenomenon was first discovered in the 1930s^10^. In 1956, Marcus and colleagues created a unified understanding of electric charge transfer theory and predicted the existence of the inverted region^11^. Later, the existence of the inverted region was demonstrated experimentally by Miller and Closs^12^. Because of its low bandgap, which can effectively increase light absorption, a BT-PC_61_BM molecule system was chosen for our calculations. In this paper, the photoinduced charge transfer rate and energy transfer mechanism of a BT-PC_61_BM molecule system were studied based on Marcus theory. We visualized the process of charge transfer using three-dimensional (3D) visualization technologies and uncovered the charge transfer and energy transfer mechanism of the optical functional materials^13^,^14^,^15^,^16^,^17^.

## Results

### Properties of the excitation state

**Figure 1:**
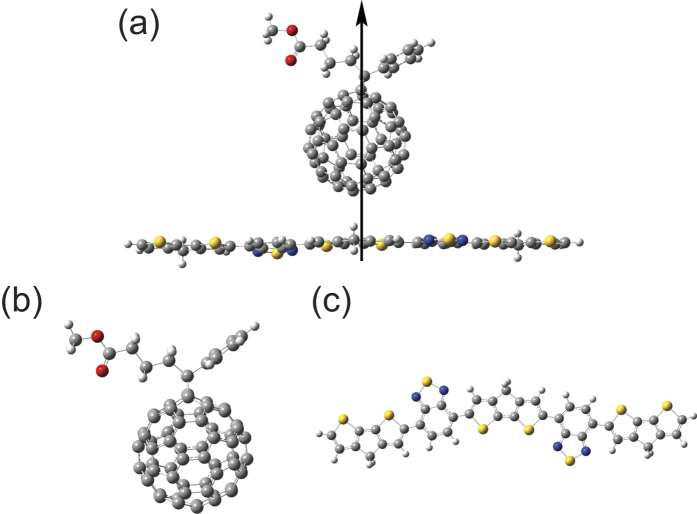
Chemical structure of the BT: PC_61_BM blend (a), PC_61_BM (b) and BT (c). The electric field is oriented along the coordinate.

A C_60_ molecule, also known as a buckyball, consists of 60 carbon atoms and has a good electron affinity. A BT molecule (see Fig. 1), with an absorption region ranging from 300 to 800 nm, mainly consists of methine (=CH-). Owing to its broad spectral response range, a BT molecule has a strong ability to capture light. In this study, we combined a BT molecule and a PC_61_BM molecule to investigate the properties of a BT-PC_61_BM molecular complex in an excited state.

**Figure 2:**
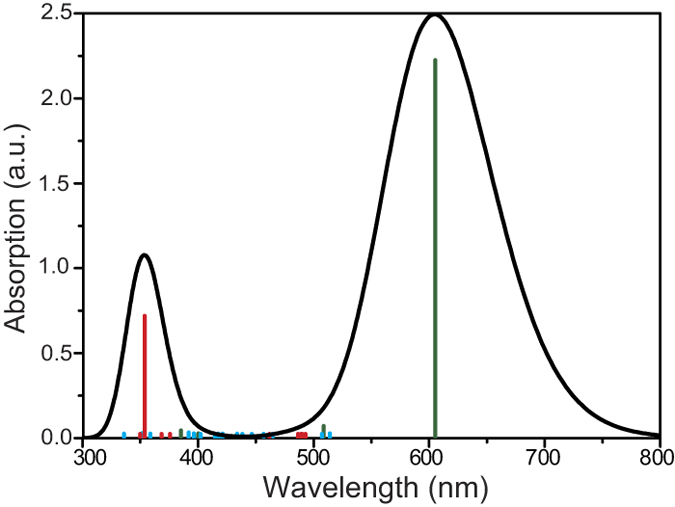
Optical electronic state absorption spectra of BT-PC_61_BM, in which the green line, red line and blue line denote LE(BT), ICT and LE(PC_61_BM), respectively.

To simulate the optical properties of a BT-PC_61_BM molecule, the excited state electronic transitions were calculated using the time-dependent density functional theory (TDDFT) method, and a long-range-corrected functional (CAM-B3LYP) was employed for the non-Coulombic part of the exchange functional. The relevant results for the calculated absorption spectroscopy of the BT-PC_61_BM molecule are shown in Fig. 2. The BT-PC_61_BM molecule had two clear absorption peaks at 605 and 353 nm. The main absorption band was in the range 300 to 800 nm, demonstrating strong absorption of visible light. The vertical lines indicate the oscillator strength of BT-PC_61_BM, whereas the blue lines express the intramolecular electric charge transfer in PC_61_BM and the red lines express the intermolecular electric charge transfer between the BT molecule and PC_61_BM molecule. Furthermore, the intermolecular electric charge transfer occurs in the range 300 to 400 nm, and an absorption peak approximately 600 nm arises from the action of the BT molecule. Table 1 lists the vertical excitation energies and oscillator strengths for the thirty excited states, as calculated using the TDDFT method based on the optimized ground-state structure of the BT-PC_61_BM molecule, and these data also support our conclusion. The first four excited states of BT-PC_61_BM correspond to intramolecular electric charge transfer, whereas the fifth and sixth excitation states correspond to electric charge transfer between the BT molecule and the PC_61_BM molecule. In this study, we focused on the intermolecular electric charge transfer associated with the fifth and sixth excitation states.

**Figure 3:**
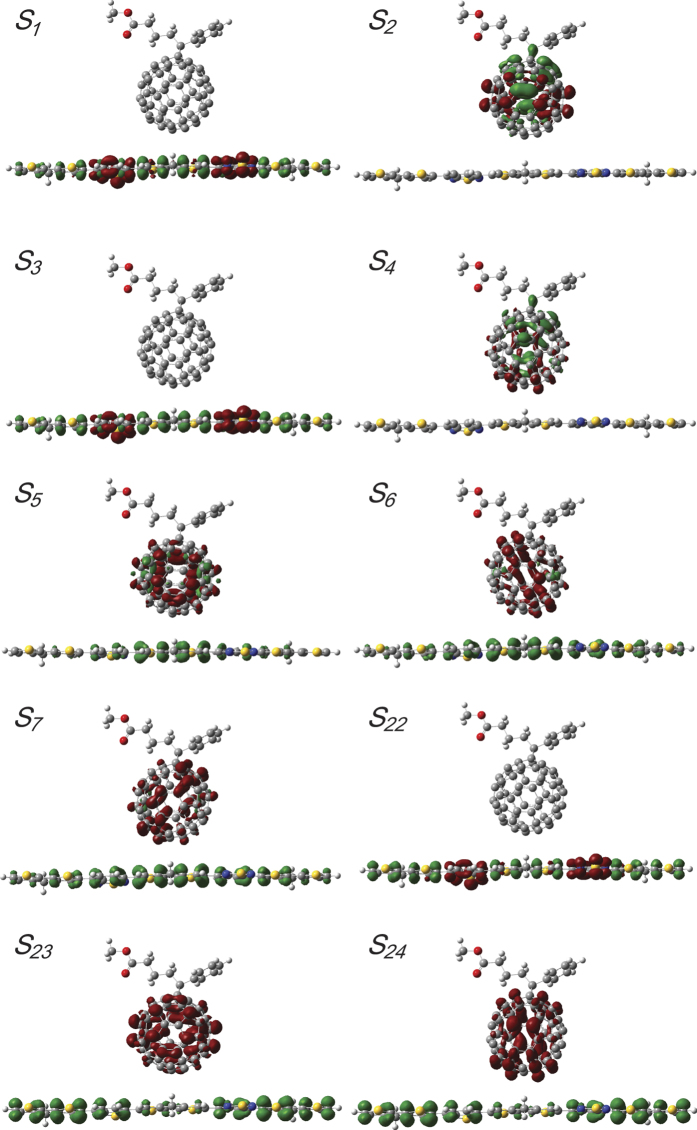
Selected charge difference densities (CDD) of the BT: PC_61_BM blend, in which the green and red colours represent the holes and electrons, respectively.

The 3D real-space analysis method has been used to analyse the charge transfer in conjugated polymers because the charge difference density (CDD) determines the orientation and results of the charge transfer in molecular and molecular-metal systems^13^. As shown in Fig. 3, two types of excited state exist in the absorption band of BT-PC_61_BM: one is the strong resonance charge transfer (CT) excited state, in which electron transfer occurs in the strong absorption region, and the other is weak resonance charge transfer. *S*_*22*_ and *S*_*23*_ are strong absorption excited states. *S*_*23*_ and *S*_*24*_ are pure intermolecular electric charge transfer excited states in which the electrons are mainly localized in the PC_61_BM molecule, and the holes are mainly localized in the main chain of the BT molecule, which demonstrates that electrons will be completely transferred from the BT molecule to the C_60_ molecule. *S*_*4*_, *S*_*5*_, *S*_*6*_ and *S*_*7*_ are weak absorption excited states. In the *S*_*4*_ excited state, the electric charge is redistributed in the PC_61_BM molecule. The electrons and holes are mainly located in C_60,_ which demonstrates that the *S*_*4*_ excited state is a localized excited (LE) state. For the *S*_*5*_ and *S*_*6*_ excited states, electrons are mainly transferred from the main chain of the BT molecule to C_60_. However, states *S*_*5*_ and *S*_*6*_ are not pure intermolecular electric charge transfer excited states, as some of the holes are still located in C_60_. Nevertheless, we are certain that *S*_*5*_ and *S*_*6*_ are the lowest intermolecular electric charge transfer states.

Figure 3 shows the qualitative visualization analysis with 3D charge difference density for charge transfer. To provide quantitative analysis of the charge transfer, Δ*r* is introduced to measure charge-transfer length^18^,


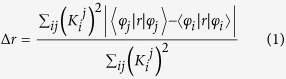


where *i* and *j* traverse all of the occupied and virtual molecular orbitals, respectively, and *φ* is the orbital wave function. The larger Δ*r* is the stronger charge transfer. The calculated results are summarized in Table 1, in which the *S*_*5*_ and *S*_*6*_ states have larger charge-transfer lengths, 3.497 Å and 4.502 Å, respectively. These calculations, along with the qualitative visualization analysis with 3D charge difference density, justify the previous conclusion that the excited-states *S*_*5*_ and *S*_*6*_ are the lowest intermolecular electric charge transfer states. The excited state *S7* (with *S6*) is degenerate and is also an intermolecular charge transfer excited state.

**Figure 4:**
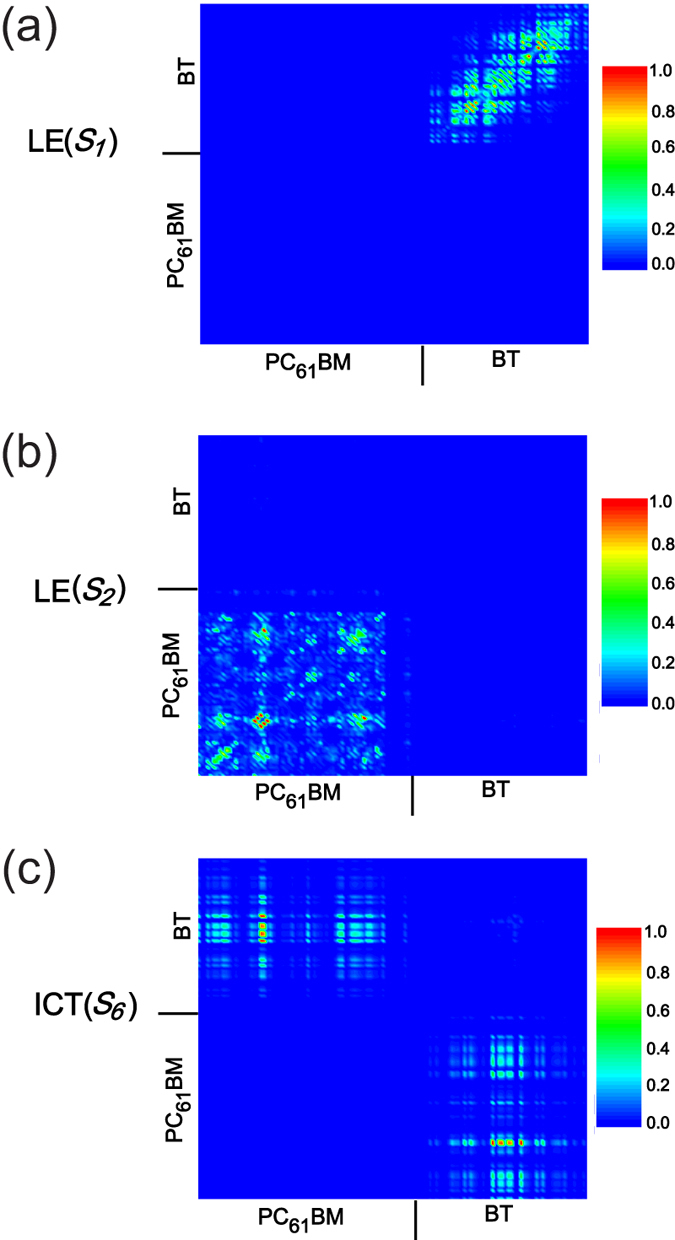
2D site representation of transition density matrixes for S_1_, S_2_ and S_6_. A colour scale bar is shown on the right.

Charge difference density and Δ*r* can be used to qualitatively and quantitatively analyse the charge transfer properties of the complex molecule in this study, but the electron-hole coherence on electronic transition cannot be demonstrated. In the two-dimensional (2D) site representation, photoexcitation creates an electron-hole pair or exciton by moving an electron from an occupied orbital to an unoccupied orbital. Each element of the transition density matrix reflects the dynamics of an exciton projected on a pair of atomic orbitals, indicated by indices, and increases the probability of finding one charged particle on site x and the second one on site y. The number of charged particles reflects the strength of the coherence between the donor and acceptor, which is defined by different colours of the element^15^. Figure 4 visualizes the electron-hole coherences, spatial span and primary sites of electron transitions. For *S*_*1*_ and *S*_*2*_, the electron-hole coherences are strong in the BT molecule and PC_61_BM, respectively. They are the π-π* transition of the inner donor and acceptor, as the electrons and holes are all localized in those units (see Fig. 4a,b). Therefore, *S*_1_ and *S*_2_ should be categorized as LE states. Furthermore, *S*_*6*_ is an intermolecular charge transfer excited state owing to the electron and hole coherence between BT and PC_61_BM. In other words, the electrons strongly cohere with holes between the the inner region of the BT molecules and the outer left region of PC61BM inner BT and left outer PC_61_BM, but this coherence is weak between the the inner region of the BT molecule and the outer right region of PC61BM (see the 2D transition density matrix in Fig. 4c).

### The charge transfer integral (electronic coupling matrix)

**Figure 5:**
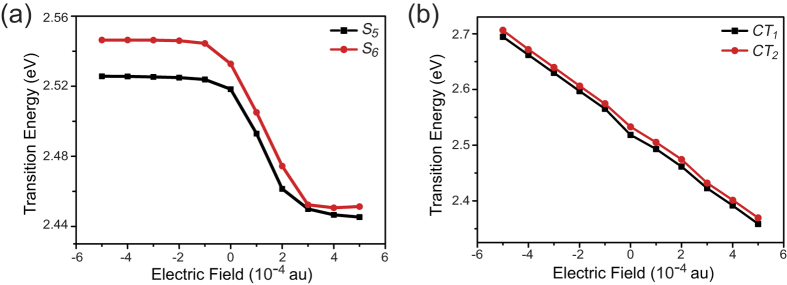
Excitation energy plotted against electric field strength for the lowest singlet excitation in PC_61_BM-BT. The squares represent calculated values. The lines are fits to Eq. 9.

The electronic coupling strength directly influences the electron transfer rate and determines the method of electron transfer. In this study, we used the generalized Mulliken-Hush (GMH) method to calculate the electronic coupling strength^19^. The expression is written as


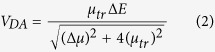


where *μ*_*tr*_ is the transition dipole moment, Δ*μ* is the difference in the dipole moments between the *S*_*0*_ and *S*_*n*_ states, and Δ*E* is the vertical excitation energy. Δ*μ* can be obtained by the finite electric field method^20^,^21^. The external electric field ***F***_ext_ is written as


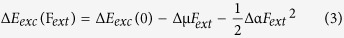


where *E*_*exc*_*(0) *= Δ*E *= *E*_*j*_*–E*_i_ is the excitation energy at the zero field and Δ*α* is the polarizability. There is widespread consensus that hyperpolarizabilities can be simulated by using the quadratic response theory method. This approach was widely used for studying two-proton absorption (TPA) and to investigate the nonlinear optical (NLO) properties of nonlinear optical materials^22^,^23^,^24^,^25^,^26^,^27^. An experimental aspect, second harmonic generation (SHG), has been used to study photoinduced charge separation dynamics in organic semiconductor thin films using the time-resolved spectra technique. Some meaningful results have been obtained, including charge separation from a gradient in excitation density and differential electron/hole mobility in model systems of fullerene (C70) and semiconductor interfaces^28^,^29^. However, it is reasonable to believe that using quadratic response theory will not considerably affect our qualitative results for photoinduced charge transport efficiency controlled by external electric field. Therefore, the second harmonic generation (SHG) effect was not considered with the time-dependent density functional method. Figure 5 shows a nonlinear effect for *S*_*5*_ and *S*_*6*_, which are the lowest intermolecular electric charge transfer excited states. The data were obtained with the excited state electronic transitions energies calculation for the BT-PC_61_BM molecular system using the TDDFT method, and the difference in the dipole moments was obtained by fitting the calculated results of the *S*_*5*_ and *S*_*6*_ excited states with Eq. (3). Our calculations show the lowest intermolecular electric charge transfer state changes when we increase or decrease the external electric field. When the external electric field is ***F***_ext_ = 1 × 10^−4^ au, the lowest intermolecular electric charge transfer state is the *S*_*5*_ or *S*_*6*_ excited state. As the external electric field increases to 5 × 10^−4^ au, the lowest intermolecular electric charge transfer excited state changes to the *S*_*2*_ or *S*_*3*_ excited state. As the external electric field decreases to −5 × 10^−4^ au, the lowest intermolecular electric charge transfer excited state changes to the *S*_*8*_ or *S*_*9*_ excited state. We first visualized and analysed the excited states under different external electric fields to determine the lowest intermolecular electric charge transfer states; then, we determined the fit for the lowest intermolecular electric charge transfer states (see Fig. 5(b)). According to Eq. (3), the differences in dipole moment (Δ*μ*) for *CT*_*1*_ and *CT*_*2*_ are 12.4427 au and 12.4364 au, respectively. For the *CT*_*1*_ excitation state, where *μ*_*tr*_ is 0.0300 au, the calculated *V*_*DA*_ is 0.0005 au. For the *CT*_*2*_ excitation state, where *μ*_*tr*_ is 0.1000 au, the calculated *V*_*DA*_ is 0.0015 au.

### Rate of exciton separation and electric charge recombination

The exciton separation and charge recombination are two separate excited-state processes, which compete with each other and together determine the photoelectric transformation efficiency of organic solar cells materials of equal importance. The free energy (Δ*G*) of exciton separation is expressed as Δ*G*_*CT*_, and the free energy of electric charge recombination is expressed as Δ*G*_*CR*_^30^,^31^,^32^,^33^,^34^,^35^,^36^. ΔG_CR_ is written as


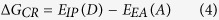


where *E*_*IP*_ *(D)* is the ionization potential of electrons in a donor and *E*_*EA*_*(A)* is the ionization potential of holes in an acceptor. The calculated *E*_*IP*_*(D)* and *E*_*EA*_*(A)* are the energies of the highest occupied molecular orbital (HOMO) and lowest unoccupied molecular orbital (LUMO) of the donor and acceptor, respectively. These quantities are normally estimated from the geometry optimization of the isolated PC_61_BM adduct and BT by using the DFT method. The calculated Δ*G*_*CR*_ is −1.5060 eV. Δ*G*_*CT*_ can be obtained by the Rehm-Weller equation:


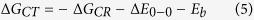


where Δ*E*_*0-0*_ is the lowest excited state energy of a free radical donor and *E*_*b*_ is the exciton binding energy. The exciton binding energy is taken as the difference between the electronic and optical band gap energies. The electronic band gap can be approximated as the energy difference of HOMO and LUMO. The calculated *E*_*b*_ is 0.2685 eV and Δ*G*_*CT*_ is −0.7686 eV. The negative value of Δ*G*_*CT*_ means that electron transfer is thermodynamically favourable.

**Figure 6:**
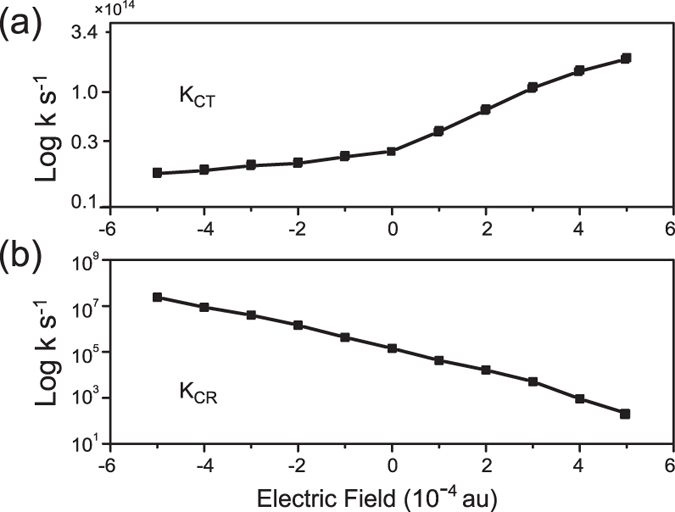
Calculated rate of exciton dissociation and charge recombination of the lowest ICT state of the PC_61_BM: BT blend with different external electric fields based on Marcus theory.

We used the Marcus model to calculate the rate of exciton separation and charge recombination, respectively. Figure 6(a) shows the calculated exciton separation rate for BT-PC_61_BM. The exciton separation rate increases by one order of magnitude with the external electric field. According to the above calculations, the increase in exciton separation occurs due to the increase in Δ*G*_*CT*_and *λ*_*CT*_ caused by the increase in the external electric field. From previous studies, we know that during the formation of the charge-separated state (D^+^A^−^), the Coulomb attraction pulls the charges back together to undergo first-order geminate recombination^37^,^38^, that is the electron and hole may be trapped in each others’ Coulomb well and forces the electric charge to recombine from the electric charge separation state at longer timescales. Also, this electric charge recombination process may occur again during exciton transport. As shown in Fig. 6(b), the change in the charge recombination rate calculated by the Marcus model with an external electric field is different from the exciton separation rate. The charge recombination rate decreases linearly with increases in the external electric field. By comparing Fig. 6(a,b), one can clear find that the initial CT dissociation rate is much larger than the recombination rate. And the relative slowness of the charge recombination rate originates from the vanishingly small electronic couplings and because recombination occurs deep in the inverted Marcus region (|Δ*G*_*CR*_| = 1.5060 eV ≫ λ_CR_ = 0.4882 eV, at ***F***_*ext*_ = 0). Additionally, the charge recombination rate decreases by 6 orders when the external electric field changes from −5 × 10^−4^ au to 5 × 10^−4^ au, which is larger than exciton separation rate. The calculated charge separation rate variations are quite small for typical electric field values for organic solar cells. This result agrees with recent charge separation studies using time-resolved electric-field induced second harmonic generation^39^. The researchers concluded that the influence of the external field on the initial charge separation was minor and that the external electric field mainly influenced the charge collection via high carrier mobility in polymers^40^.

Finally, the hot charge transfer exciton mechanism in organic photovoltaics may offer an alternative explanation for our computational results that the exciton separation rate is much higher than that of charge recombination^41^. The excess energy from charge separation results in a charge pair at an initial distance in the Coulomb potential. If this initial distance is longer than the critical escape distance, where the Coulomb binding energy is less than the thermal activation energy, charge separation occurs. Otherwise, the charge pair relaxes towards contact and probably eventual recombination^5^. For polymer/fullerene interfaces, the excess energy of hot CT excitons may still play a role in assisting charge separation, even at sufficiently low temperatures^42^. This is mainly because hot CT excitons have relative weaker bonds from the Coulomb potential, and these excitons can be easier to dissociate. Electronic coupling from a donor exciton to a hot charge transfer exciton across the D/A interface can also be higher than the normal charge transfer expected from energy resonance. As a result, hole delocalization can further reduce the Coulomb attraction effect and accelerate the charge transfer process^43^. According to the results of our theoretical simulation on the BT-PC_61_BM complex, the calculated *E*_*b*_ is 0.2685 eV and Δ*G*_*CT*_ is −0.7686 eV respectively, excitons can sufficiently separate into electrons and holes at ***F***_*ext*_ = 0 from the interfacial hot CT excitons perspective. Therefore, for this photoactive material in organic solar cells, the excitons separate into electrons and holes before they arrive at the donor-acceptor interface. ***But, according to our results, we must state that exciton dissociation is not the limiting factor in organic solar cells.***Noted that, whether the interfacial energy gradient can overcome the Coulomb trap or not clearly should be system specific. These conclusions can greatly improve the photoelectric transformation efficiency of organic solar cells and decrease the loss of electrons in the transport process. Our findings are in accordance with a recent study revealing the enhanced probability of charge separation from highly delocalized hot interfacial charge transfer states^44^, and provide an important design principle for new organic photovoltaics materials.

## Discussion

In this study, the excited states of BT and PC_61_BM molecules were examined with a quantum-mechanical method. The BT-PC_61_BM complex has a broader spectrum response region than regular response regions, and strong absorption peaks are located in the visible light range. The lowest electric charge transfer state changes as the external electric field changes. The BT-PC_61_BM complex molecule has a high electric charge separation rate (3.1334 × 10^13^ s^−1^) and a low electric charge recombination rate (1.4221 × 10^5^ s^−1^) according to the Marcus model at ***F***_ext_ = 0. After adding an external electric field, the charge recombination rate calculated with the Marcus model decreased linearly as the external electric field increased. The charge recombination rate decreased by 6 orders when the external electric field increased from −5 × 10^−4^ au to 5 × 10^−4^ au. The exciton separation rate increased with increases in the external electric field. The exciton separation rate is increased by one order. ***But, the exciton dissociation is not the limiting factor in organic solar cells according to our results.***

## Methods

### Charge transfer rate

For nonadiabatic and adiabatic reactions, the charge transfer is handled using different approximations. We chose the classic Marcus mode. In classic Marcus theory, Marcus proposed three assumptions about an adiabatic reaction: 1) The reactants have the same energy as the products, and the energy is conserved before and after the state-to-state transition; 2) The Frank-Condon principle is met; 3) The energy split is large, and the transition probability is 1 at the position of the transition state^45^.

Exciton dissociation and charge recombination relate to the electron transition reaction. In the semi-classical limit from Marcus theory, the charge transfer rate is expressed as


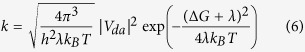


where *k*_*B*_ is the Boltzmann constant, *h* is Planck’s constant, *V*_*da*_ is the electronic coupling between the initial and final states, *λ* is the reorganization energy, and *T* is the temperature (*T *= 300 K).

### Reorganization energy

The reorganization energy is an important parameter for characterizing the electron and energy transfer. According to the rate expression (Eq. (6)), the rate reaches its maximum value when the reorganization energy is at its minimum value^46^,^47^,^48^. In our research, we focused on the relationship between the inner reorganization energy and electron transfer. Before and after the electron transfer, the entire system will relax at a new steady state. The dissipative energy in the relaxation process is the reorganization energy. In other words, the reorganization energy is the relaxation energy. The reorganization energy is written as


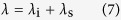


where *λ*_*i*_ is the internal reorganization energy arising from the change in equilibrium geometry of the donor and acceptor sites upon electron transfer and *λ*_*s*_ is the outer reorganization energy.

The inner reorganization energy consists of two sections^49^,^50^:


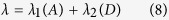



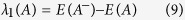



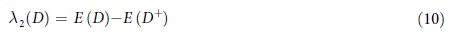


where *E(A*^−^) and *E(A)* are the energies of the neutral acceptor A at the anion geometry and the optimal ground-state geometry, respectively, and *E(D)* and *E(D*^+^) are the energies of the radical cation at the neutral geometry and optimal cation geometry. *λ*_*s*_ in Eq. (7) is the outer reorganization energy, which is related to the change in electronic and nuclear polarizations in electron transfer. The outer reorganization energy can be expressed as


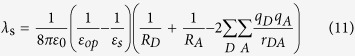


where *R*_*D*_ and *R*_*A*_ are the radii of the donor and acceptor, respectively. *q*_*D*_ and *q*_*A*_ are the electric charge of the donor and acceptor, respectively. *r*_*DA*_ is the donor-acceptor distance. *ε*_*0*_ is the dielectric constant in a vacuum. *ε*_*op*_ is the optical dielectric constant of the surrounding media and *ε*_*s*_ is the relative dielectric constant of the molecule^51^,^52^,^53^,^54^.

### Quantum chemical calculation method

BT and PC_61_BM were chosen as the photoactive materials for organic solar cells. All quantum chemistry calculations were performed by Gaussian 09 software^55^. The ground-state geometries of the BT-PC61BM molecule, isolated PC_61_BM adduct and BT were optimized using density functional theory (DFT)^56^, the B3LYP functional^57^,^58^, and the 6-31G(d) basis set. The molecular structures of isolated BT and PC_61_BM are shown in Fig. 1. To calculate the reorganization energies of the charge transfer reaction in Marcus theory, the cationic ground state geometry of isolated BT and the anionic ground state geometries of the isolated PC_61_BM adduct were also optimized with DFT, the B3LYP functional, and the 6-31G(d) basis set. Then, the single point energies of these two neutral acceptors at the anionic geometry and optimal ground-state geometry, as well as the single point energies of the radical cation at the neutral geometry and optimal cation geometry, were calculated at the same level of theory. Although previous theoretical studies have revealed that the conventional hybrid B3LYP functional could be sufficiently accurate for calculating charge transfer excited-states in some systems^59^,^60^, the long-range-correction should be considered in quantum chemical calculations of large systems, such as the organic solar cell donor-acceptor heterojunction in this study^61^. Therefore, to simulate the optical absorption properties, a calculation of the excited state electronic transitions BT-PC_61_BM molecular system was performed using time-dependent density functional theory (TDDFT)^62^, the long-range-corrected functional (CAM-B3LYP)^63^, and the 6-31G(d) basis set. The long-range-corrected functional was employed for the non-Coulombic part of the exchange functional. Furthermore, geometry optimization of the lowest excited state of the isolated donor and the lowest excited state of the radical cation state was performed with TD-DFT, CAM-B3LYP, and the 6-31G (d) basis set. The *Generalized Mulliken*-*Hush* (GMH) model was employed to calculate the charge transfer integral (electronic coupling matrix). To investigate the effect of an external electric field on the excited-state properties of the molecules, the finite field method was employed, and the direction of the electric field is shown in Fig. 1. Fields ranging from −5 × 10^−4^ to 5 × 10^−4^ au were used. This result can be compared to the realistic strength of the electric field in the solar cell devices of up to 4 × 10^−5^ au (∼2 × 107 V/m). The field in a solar cell can be oriented in all possible directions. To visualize charge transfer during electronic transitions, two-dimensional (2D) site space analysis (transition density matrix) and three-dimensional (3D) real space analysis (charge difference density) were performed, which were described in detail in our previous article^13^.

## Additional Information

**How to cite this article**: Li, Y. *et al.* Photoinduced Charge Transport in a BHJ Solar Cell Controlled by an External Electric Field. *Sci. Rep.* **5**, 13970; doi: 10.1038/srep13970 (2015).

## Figures and Tables

**Table 1:** Selected electronic transition energies (eV) and corresponding oscillator strengths (*f*), main compositions and CI coefficients of the BT: PC_61_BM blend.

**States**	**Transition Energy (eV)**^a^	***f***^b^	**Δr(Å)**^c^	**Excited-state Property**^d^
*S*_*1*_	2.0506 (604.63 nm)	2.2006	0.759739	LE
*S*_*2*_	2.4156 (513.27 nm)	0.0024	0.598960	LE
*S*_*3*_	2.4404 (508.06 nm)	0.0467	0.419422	LE
*S*_*4*_	2.4476 (506.55 nm)	0.0000	0.753877	LE
*S*_*5*_	2.5183 (492.33 nm)	0.0001	3.497080	ICT
*S*_*6*_	2.5328 (489.52 nm)	0.0006	4.502078	ICT
*S*_*7*_	2.5382 (488.47 nm)	0.0006	5.387317	ICT
*S*_*8*_	2.5523 (485.78 nm)	0.0000	1.796438	ICT
*S*_*9*_	2.6739 (463.68 nm)	0.0001	0.178010	LE
*S*_*10*_	2.6891 (461.06 nm)	0.0001	0.569387	LE
*S*_*11*_	2.7191 (455.97 nm)	0.0004	0.619133	LE
*S*_*12*_	2.7810 (445.83 nm)	0.0000	0.505284	LE
*S*_*13*_	2.8326 (437.70 nm)	0.0000	0.199343	LE
*S*_*14*_	2.8624 (433.15 nm)	0.0009	0.665378	LE
*S*_*15*_	2.9473 (420.68 nm)	0.0014	0.706160	LE
*S*_*16*_	2.9740 (416.90 nm)	0.0013	1.097320	LE
*S*_*17*_	2.9945 (414.05 nm)	0.0000	0.601590	LE
*S*_*18*_	3.0872 (401.61 nm)	0.0003	0.777263	LE
*S*_*19*_	3.1032 (399.53 nm)	0.0026	0.735057	LE
*S*_*20*_	3.1329 (395.75 nm)	0.0025	0.794401	LE
*S*_*21*_	3.1703 (391.09 nm)	0.0081	0.158442	LE
*S*_*22*_	3.2247 (384.49 nm)	0.0200	0.719405	LE
*S*_*23*_	3.3056 (375.07 nm)	0.0000	7.582522	ICT
*S*_*24*_	3.3712 (367.78 nm)	0.0006	7.344502	ICT
*S*_*25*_	3.4619 (358.14 nm)	0.0012	0.338456	LE
*S*_*26*_	3.5109 (353.14 nm)	0.2450	0.800606	LE
*S*_*27*_	3.5121 (353.02 nm)	0.6954	1.791835	LE
*S*_*28*_	3.5327 (350.96 nm)	0.0037	1.127917	LE
*S*_*29*_	3.5501 (349.24 nm)	0.0005	6.733170	ICT
*S*_*30*_	3.6982 (335.25 nm)	0.0013	0.230103	LE

^a^The numbers in parentheses are the transition energy wavelength.

^b^Oscillator strength.

^c^Δr index is a quantitative indicator of electron excitation mode, which is a measure of CT length.

^d^PC_61_BM and BT in parentheses present the density are localized on the fullerene and polymer, respectively.
